# Discovering gene expression signatures responding to tyrosine kinase inhibitor treatment in chronic myeloid leukemia

**DOI:** 10.1186/s12920-016-0194-5

**Published:** 2016-08-12

**Authors:** Kihoon Cha, Yi Li, Gwan-Su Yi

**Affiliations:** Department of Bio and Brain Engineering, KAIST, Daejeon, 34141 South Korea

**Keywords:** Gene expression signature, Chronic myeloid leukemia (CML), Tyrosine kinase inhibitor (TKI), Meta-analysis, Random forest

## Abstract

**Background:**

Tyrosine kinase inhibitor (TKI)-based therapy is a recommended treatment for patients with chronic myeloid leukemia (CML). However, a considerable group of CML patients do not respond well to the TKI therapy. Challenging to overcome this problem, we tried to discover molecular signatures in gene expression profiles to discriminate the responders and non-responders of TKI therapy.

**Methods:**

We collected three microarray datasets of CML patients having total 73 responders and 38 non-responders. Statistical analysis was performed to identify differentially expressed genes (DEGs) as gene signature candidates from integrated microarray datasets. The classification performance of these genes and further selected discriminator gene sets was tested by using random forest and iterative backward variable selection methods.

**Results:**

We identified a set of genes including CTBP2, NADK, AZU1, CTSH, FSTL1, and HDLBP showing the highest accuracy more than 69.44 % to classify TKI response in CML patients. Interestingly, four genes of them are on the signaling pathway of cell proliferation. This set of genes showed much higher performance than the average performance of other genes in downstream signaling of TKI target, BCR-ABL.

**Conclusions:**

In this study, we could find a set of potential companion diagnostic markers for TKI treatment and, at the same time, the potential of gene expression analysis to enhance the coverage of companion diagnostics.

**Electronic supplementary material:**

The online version of this article (doi:10.1186/s12920-016-0194-5) contains supplementary material, which is available to authorized users.

## Background

Chronic myeloid leukemia (CML) is a myeloproliferative disease with pluripotent hematopoietic cell and caused by a reciprocal translocation between chromosome nine and chromosome 22, which is specifically designated t(9;22)(q34;q11) [1]. This translocation creates a novel fusion gene, BCR-ABL, which encodes a constitutively active isoform of ABL tyrosine kinase (TK) and leads to pathophysiology of CML [2–5]. Treatment with tyrosine kinase inhibitor (TKI) such as Imatinib, Dasatinib, and Nilotinib had been proved to be an effective therapy as inducing a complete cytogenetic response in more than half of with newly CML patients [6, 7]. However, a lot of patients failed to TK inhibitor treatment because of intrinsically resistant or developed resistance to drugs [8]. In order to increase efficiency of treatment, it is necessary to predict the response to drugs which patients would benefit from treatment before clinical therapy.

DNA Microarray is one of the most powerful technology developed in recent years to profile gene expression, identifying the differentially expressed genes (DEGs), correlation of genes and their biological pathways [9–12]. DNA microarray and following data analysis solutions have become a new research tool for a disease diagnosis, prognosis, monitoring progress of a disease, and discovering gene signatures of various diseases [13, 14]. For example, based on multiple microarray data indicating drug response condition from RA patients, common DEGs were found in different dataset and one of them was selected as most believable biomarker by meta-analysis method [14]. In the aspect of cancer, patient classifier was set up based on microarray data from Imatinib-naive CML patients and correctly predicted responders and non-responders [15]. In addition, besides protein-encoding gene, long noncoding RNAs (lncRNAs) were found significantly changed between Dasatinib-resistance/sensitive patients, which indicated lncRNAs might be related to mechanisms of drug response [16]. Although DEG sets were identified from each dataset, it is necessary to integrate them and to identify gene expression signatures to predict the drug response with a more reliability in inter-patient heterogeneity.

To this end, we compiled three microarray datasets from CML patients with the clinical outcome of TKI therapy. Therefore, we used statistical analysis to identify DEGs as gene signature candidates from three sets of microarray datasets covering 101 CML patients grouped by the response of TKI treatment. After statistical analysis on gene expression profiles, we selected the gene signatures to discriminate responder and non-responder patients treated with TKI agents using a random forest (RF) classifier. In addition, we performed functional annotation of these gene signatures to figure out the role of TKI related pathway in CML. We found that four genes were associated with cell proliferation of TKI resistance mechanisms in CML. This study provided to develop a robust gene expression signature-based classifier of the clinical outcome to TKI-based therapy. Moreover, our finding suggests biomarker candidates that could discriminate responder and non-responder patients treated with TKI. It would help to apply companion diagnostics by further experimental validation of putative biomarkers and to discover key targets of novel drugs for patients.

## Methods

### Collection of microarray data

We searched microarray dataset to find available gene expression profiles that could predict treatment outcome of TKI therapy in CML patients. Microarray data were derived from the NCBI Gene Expression Omnibus (GEO) web site by KEY words such as “Imatinib”, “Dasatinib”, “Drug Response”, “Gene Expression”, and “Chronic Myeloid Leukemia” as dataset title and descriptions. We focused on the microarray data from blood samples with responder and non-responder patient treated with drugs targeting the same target because we were interested in collection of multiple microarray data to provide validated conclusion.

We selected three sets of gene expression datasets: GSE14671, GSE2535, and GSE33224. The GSE14671 dataset included 41 blood samples from responder patients and 18 samples from non-responder patients to Imatinib. This dataset was based on the Affymetrix Human Genome U133 Plus 2.0 Array [15]. The GSE2535 dataset included 16 blood or bone marrow samples from responder patients and 12 samples from non-responder patients to Imatinib; this dataset was based on the Affymetrix Human Genome U95 Version 2 Array [6]. The GSE33224 dataset included 12 peripheral blood samples from responder patients treated with Dasatinib, 16 samples from non-responder patients measured on Agilent-014850 Whole Human Genome Microarray 4x44K G4112F [16]. SOFT formatted family files of three sets of microarray data were parsed by using the GEOquery R package [17]. We collected the 101 samples including 73 responders and 38 non-responders from three gene expression datasets. The 101 samples were randomly selected 38 responders and 38 non-responders for avoiding overfitting. Next, 76 samples were divided into two-thirds training and one-third testing datasets for gene signature selection and performance test of them.

### Microarray data preprocessing

With two-channel microarray dataset GSE33224, we firstly combined two dye swap technical replicates into one by take the average of them and transformed the expression values by inverting the log2-transformation. Then, we processed quantile normalization to each of the three microarray datasets using the limma R package [18]. We converted the probe IDs into Entrez Gene IDs using platform information of each dataset to make the unique ID. We mapped to the Entrez Gene IDs from probe IDs in each set of microarray data and collapsed their expression values by averaging them to make each microarray dataset contain non-redundant set of genes.

### Selection of gene signature candidates using statistical analysis

We analyzed each microarray dataset individually to identify gene signatures that are differentially expressed in two conditions of responder and non-responder patients treated with TKI. Student *t*-test analysis coupled with False Discovery Rate for multiple testing corrections were performed using the genefilter R package [19] to find out the DEGs between responders and non-responder groups.

We performed meta-analysis to combine the results of each microarray dataset and to extract more robust DEGs. We used MetaQC and MetaDE in R packages for quality controls and DEGs identification [20]. MetaQC calculated six quantitative quality control (QC) measures: internal quality control for homogeneity of co-expression structure among studies (IQC), external quality control for consistency of co-expression pattern with pathway database (EQC), and accuracy and consistency quality control of differentially expressed gene detection (AQCg and CQCg) or enriched pathway identification (AQCp and CQCp). MetaDE contained 12 major meta-analysis method for DEG detection including three categories of combining *P*-value, combining effect size, and combining ranks. After QC measure process, the dataset with a poor IQC score excluded from meta-analysis which indicates that this dataset has a heterogeneous information with other datasets. We used the moderated-t statistics as an argument of function ind.method to calculate p-values of each gene in each microarray dataset. In addition, we conducted meta-analysis that identify DEGs from the results of each dataset using MetaDE with the three popular meta-analysis methods including Fisher method, maximum p-value (maxP) method, and adaptively weighted (AW) Fisher method of a type of combining *p*-values. The Fisher method summed up minus log-transformed *p*-values so that larger score reflected integrated differentially expressed evidence. The maxP method is taken as the maximum *p*-value among datasets. The AW Fisher method characterizes effective studies contributing to the meta-analysis for better biological interpretation.

### Identification of gene signatures with random forest

To identify gene signatures for discriminating patients between responders and non-responders, we performed classification analysis either responders or non-responders using RF. The RF algorithm is a combinational classifier that selects one classifier model by constructing multiple classification trees. Each classification tree is constructed using bootstrap sample of two-thirds of datasets from total datasets. RF method has several properties that are less overfitting, feature selection, and robust performance by parameter choices. RF model was selected to find sets of gene signatures by Gini variable importance and was evaluated by out-of-bag (OOB) testing. This OOB estimate is as accurate as using validation test with a test set of the same size as the training set. To find a set of gene signatures from results of statistical analysis, RF was performed by using varSelRF [21] that can select the sets of gene signatures with high accuracy. The following arguments of varSelRF were used for selection of gene sets: ntree = 10000, ntreeIterat = 2000, mtryFactor = 1, and vars.drop.frac = 0.02. We finally selected and validated a set of gene expression signatures from testing datasets.

### Functional analysis

The functional meaning and related pathway information of the each list of DEGs that was identified by individual analysis and meta-analysis was interpreted using functional enrichment analysis. This analysis was based on a one-sided Fisher’s exact test using Gene Ontology (GO), KEGG pathway, BIOCARTA pathway, Panther pathways, and Reactome pathway in The Database for Annotation, Visualization and Integrated Discovery (DAVID) [22]. The *p*-values were adjusted by multiple testing corrections using Benjamini correction method.

## Results

### Characteristics of analyzed dataset

To perform this study, we selected three available studies that examined gene expression profiles of blood samples to predict treatment outcome of TKI therapy in CML patients. Detailed information of each dataset was described in Table 1. In the dataset of GSE2535, patients from consecutive Novartis-sponsored trials were included. Imatinib responders were defined as they achieved complete cytogenetic response (CCR) within 9 months while non-responders failed to achieve a major cytogenetic response (MCR) within 1 year of treatment. Samples from total bone marrow (BM) white cells, or peripheral blood (PB) white cells were collected to perform microarray. In GSE14671, patients who had failed in prior interferon-α-based therapy were included. Imatinib responders were defined as they showed cytogenetic response (CR) within 12 months of treatment while non-responders as all other patients. Cells from BM were collected and then CD34+ cell were selected as samples for microarray. In GSE33224, patients previously resistant to Imatinib were included. Dasatinib responders were defined as they achieved CR while non-responder belonged to ≥ 90 % Ph + metaphases. Samples from peripheral blood mononuclear cells (PBMCs) were collected to perform microarray.

**Table 1:** Studies included in analysis

Study	GEO dataset	The number of samples		Cell type/Tissue	Drug	Platform
		Responder	Non-responder			
McWeeney et al., 2010 [15]	GSE14671	41	18	CD34+ cell/BM	Imatinib	HG-U133_Plus_2
Crossman et al., 2005 [6]	GSE2535	16	12	White cell/Blood, BM	Imatinib	HG-U95Av2
Silveira et al., 2013 [16]	GSE33224	6	8	PBMC/Peripheral Blood	Dasatinib	Agilent-014850

Since previous studies were performed individually accompanying with clinical and experimental variations, as well as being analyzed by different statistical analysis methods, no overlapped DEGs were found between them. Even though, two studies set up models of drug response classifiers based on DEGs from single dataset, and one of them correctly predicted ≥80 % of responder and non-responder [15]. Another studies evaluated DEGs/lncRNAs functional relevance by using Ingenuity Pathway Analysis (IPA) tools. Interestingly, most of DEGs were identified within “Cell-to-Cell Signaling and Interaction” category and differently expressed lncRNAs were in “Cell Death” category [16].

### Selection of gene signature candidates

**Fig. 1:**
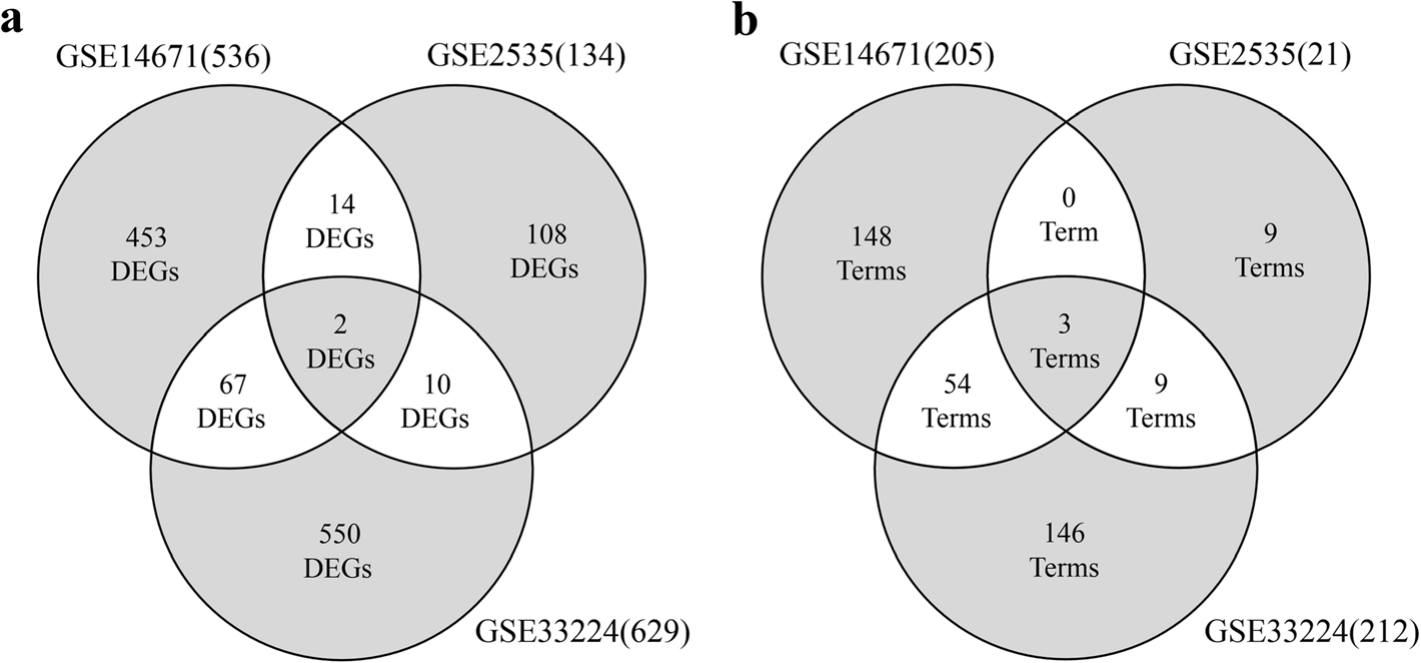
Duplicate analysis of differentially expressed genes and related functional terms for each microarray dataset. **a** Venn diagram showing the overlap of DEGs between each dataset and **b** Venn diagram showing the overlap of functional annotation terms between each dataset

To combine and compare the DEGs of each dataset, it is necessary to analysis datasets with coherent conditions. We firstly filtered 5658 probes which were examined in all datasets, extracting their values to perform following analysis steps. Then, we performed student’s *t*-test to identify DEGs between responder and non-responder groups from individual dataset. FDR as multiple-testing was used to adjust *p*-values. Since none of the genes passed the significant threshold with FDR *p*-value < 0.05, we selected DEGs based on raw *p*-values and set threshold as 0.05. We identified 536 DEGs for GSE14671, 134 DEGs for GSE2535, and 629 DEGs for GSE33224 (Additional file 1: Table S1). With comparing DEGs in 3 datasets, we found 14 DEGs overlapped between GSE14671 and GSE2535, 67 DEGs overlapped between GSE14673 and GSE33224, 10 DEGs overlapped between GSE2535 and GSE33224, and 2 DEGs (PARP3, SUMO3) overlapped in all three datasets (Fig. 1a).

To further investigate the role of DEGs, we performed functional enrichment to DEGs in each dataset by DAVID. Raw *p*-value threshold was set to 0.05 and significantly enriched annotation terms were shown in Additional file 2: Table S2. 205 annotation terms were found in GSE14671 and the most significant term, “defense response”, contains 54 genes. 21 annotation terms were found in GSE2535 and the most significant term “immune response” contains 16 genes. 212 annotation terms were found in GSE33224 and the most significant term, “response to organic substance” contains 55 genes. Duplicate analysis showed 3 overlapped annotation terms including “defense response”, “immune response” and “response to organic substance” were found among all datasets. 57 overlapped annotation terms including “immune response”, “defense response”, “response to organic substance” and “homeostatic process” were found between GSE14671 and GSE33224. 12 overlapped annotation terms including “immune response”, “defense response”, “response to organic substance” and “actin cytoskeleton organization” were found between GSE2535 and GSE33224. Eventually, three overlapped annotation terms “immune response”, “defense response” and “response to organic substance” were shared by all datasets (Fig. 1b). Considering the fact that only a few number of common DEGs and functional terms found in all datasets, we guessed individual analysis for dataset separately provide insufficient statistic power and the result is variously dependent on experimental or sample bias, limiting to find the common features from different datasets.

To overcome the problem, we used meta-analysis on gene expression profiles that combines the results from independent datasets to increase statistical power. To measure six quantitative QC, all 3 microarray datasets with 1299 DEGs according to above individual analysis were included. Although the scores of CQCg, CQCp, AQCg, and AQCp from all datasets were not statistically significant, there was none of poor score of IQC from any datasets. Therefore, we then conducted meta-analysis on all genes of the datasets. We used three *p*-value combination methods including Fisher method, maximum *p*-value method, and adaptively weighted Fisher method for meta-analysis. We identified 54 DEGs with Fisher method, 45 DEGs with maxP method, and 56 DEGs with AW method when raw *p*-value <0.01 was set as threshold (Table 2). We further investigate the biological process of these DEGs. We integrated non-redundant 99 DEGs by using three different methods and performed a functional enrichment analysis based on GO and pathway resources. 31 significant functional terms with raw *p*-value <0.05 were found that the most significant term “anti-apoptosis” with *p*-value of 1.65E-03 and other terms related with CML including “regulation of apoptosis”, “regulation of cell death”, “regulation of programmed cell death”, and “myeloid leukocyte activation” were significantly enriched.

**Table 2:** The number of differentially expressed genes by meta-analysis methods

*p*-value	Fisher	maxP	AW
*P* < 0.01	54	45	56
*P* < 0.05	203	189	212

### Identification of gene signatures

We aimed to discover an optimal set of gene signatures for patient classification with TKI-based therapy. So, we performed Random Forest (RF) with DEGs which were identified by meta-analysis above as features to select the most accurate and smallest gene signature set. In Table 3, 99 gene combinations with low OOB error were identified. Moreover, the set of six genes including CTBP2, NADK, AZU1, CTSH, FSTL1 and HDLBP, with least OOB error of 0.2291 from training dataset were selected by variable selection. Next, based on six gene signatures, we construct RF model from training set and test performance of classification by testing set. Accuracy of the six gene signatures was 69.44 %. Ten genes in both BCR-ABL downstream signaling pahtway and our datasets including GRB2, KRAS, MAPK1, PIK3CG, AKT1, AKT3, CBL, SHC1, CSK, and SRC were collected by Kolch W. and Pitt A. study [23]. As a result, seven sets of genes were selected by RF and a set of GRB2 and KRAS was the lowest OOB error rate of 0.278. In addition, average classification error of seven sets of genes was 0.321.

**Table 3:** Error rates of sets of gene signatures

Number of genes	OOB error	SD.OOB
99	0.2916667	0.06560571
79	0.2083333	0.05861786
63	0.25	0.0625
50	0.25	0.0625
40	0.2708333	0.0641422
32	0.25	0.0625
26	0.2291667	0.0606646
21	0.2291667	0.0606646
17	0.2291667	0.0606646
14	0.2291667	0.0606646
11	0.2291667	0.0606646
9	0.2291667	0.0606646
7	0.2291667	0.0606646
6	0.2291667	0.0606646
5	0.3125	0.06690225

After identifying six genes signatures, we then interested in these genes function and how they participate in CML development or TKI-resistance mechanism in patients. We surveyed canonical pathways of CML signaling and TKI signaling from literatures, IPA software and public biological databases. First of all, to find relationships between six gene signatures and known CML-associated genes, we collected 258 CML-associated genes from five available disease-associated databases including OMIM (Online Mendelian Inheritance in Man) [24], Genetic Association Database [25], PharmGKB [26], KEGG DISEASE [27], and Cancer gene census [28]. None of the six genes mapped with CML-associated genes. We then used our comprehensive protein-protein interaction database, ComBiCom [29] to discover interaction between six gene signatures and CML-associated genes. As a result, two genes, CTBP2 and FSTL1, directly interact with three proteins (BCL3, MDM2, and MDS1) and one protein (TGFB1), respectively.

On the other aspect, we investigated relationship between six gene signatures and TKI-resistance mechanisms in CML. We filtered out 22 molecules form “Imatinib-resistant CML disease” in IPA and manually added another 26 TKI-resistance related molecules which mentioned in literatures but not contained in IPA. The added genes were listed as follows: 1) 16 genes related to alternative signaling pathways, 2) four genes related drug transporter regulation, 3) two genes related to DNA repair pathway, and 4) four genes related to epigenetic modification [30, 31]. After input of six gene signatures, we expanded connections between each molecules and overlaid “disease & function” and “canonical pathway” layer to all genes. As a result, CTBP2 was found to interact and decrease activity of HDACs as well as increase activity of PI3K. In addition, CTBP2 located in the downstream of TGF-beta signal pathway in CML, mediating cell growth inhibition. PP1 was found as intermediate between AZU1 and AKT/MAPK/SRC signaling pathways. PP1 inhibits AZU1 release and reduces activation of PI3K, SRC, BCR-xL, Ras and AKT. HDLBP inhibits mRNA of CSF1R which activates PI3K, AKT, and STAT and also interacts with HDACs. In summary, three of the total six genes (CTBP2, AZU1 and HDLBP) were found to be related with TKI-resistance mechanisms.

## Discussion

We performed combined analysis with statistical analysis and classification analysis on gene expression data to identify gene expression signatures for classification of CML patients to the drug response of TKI-based therapy. Based on our results, there was a few overlapped DEGs among microarray datasets by individual analysis. It could be caused by a diverse of variables in each dataset such as characteristics of patients, platforms of microarray, analysis methods, and a type of drugs. To diminish the bias, we compared three sets of microarray data by three different method of meta-analysis and discovered 99 non-redundant DEGs by Fisher method, maxP method, and AW method with *p*-value <0.01. Among them, a set of six DEGs including CTBP2, NADK, AZU1, CTSH, FSTL1, and HDLBP with the highest accuracy of 69.44 % was identified. Moreover, we found that CTBP2, NADK, AZU1, and HDLBP were related to BCR-ABL inhibitor resistance mechanisms in BCR-ABL downstream signaling pathways.

**Fig. 2:**
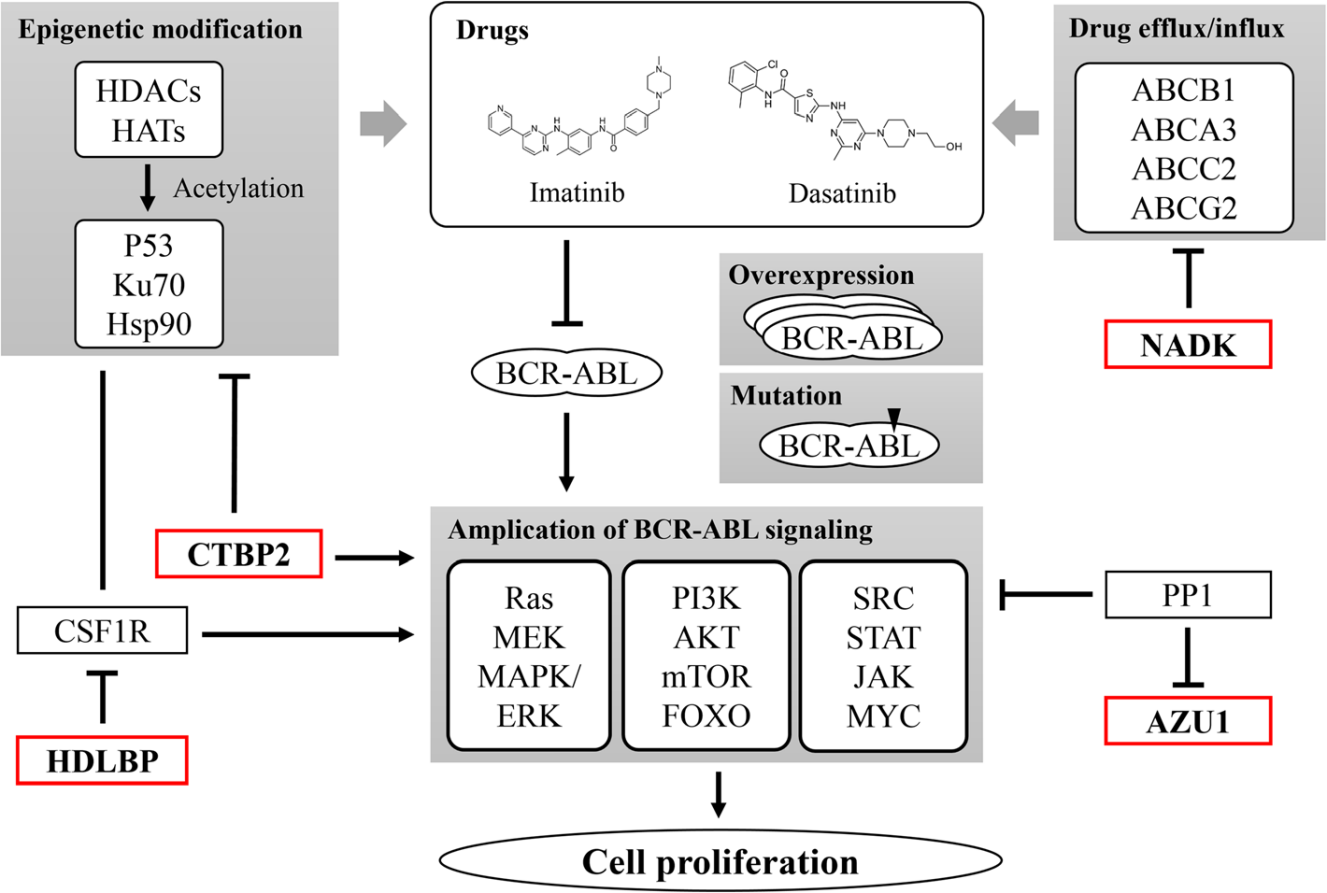
Four gene signatures (*red border*) in BCR-ABL inhibitor resistance mechanism

In CML, two group mechanisms, BCR-ABL-dependent and –independent mechanisms, have been found to contribute TKI-resistance [31]. BCR-ABL mutation in kinase domain or ATP-binding domain, BCR-ABL amplification and BCR-ABL signaling impairing belongs to BCR-ABL-dependent group whereas TKI metabolism, drug transporters-induced TKI influx/efflux, micro-environment such as inflammatory elements or hypoxia alteration and DNA repair mechanism deficient induced BCR-ABL-independent pathways [32]. With subsequently functional analysis, we identified three genes, CTBP2, HDLBP, and AZU1, related to TKI-resistance mechanism. Although FSTL1 were identified to interact with TGF beta-dependent pathway, it is still out our discussion area because it not related to BCR-ABL pathways in CML (Fig. 2). Whereas NADK was not identified by IPA, but it may also affect drug efflux/influx by completely consume ATP with TKI membrane transporter, leading to decrease efficacy of transport TKI into cells. CTBP2 is the most closely related genes since it directly interacts with downstream component of BCR-ABL signal as well as regulating HDAC and PI3K, leading to alteration of epigenetic modification and activation of PI3K/AKT signal pathway. HDLBP indirectly block BCR-ABL downstream signaling by block the expression of intermediator CSF1R. PP1, a negative intermediator for AZU1, prevents downstream signaling pathway, leading to co-changes between AZU1 and CML signaling pathways.

## Conclusions

 We performed both statistical and classification analysis to identify gene expression signatures to discriminate patients between responder and non-responder groups from integrated gene expression profiles. In our analysis, we used three gene expression profiles which were different in terms of platforms, sample sources, drugs, and clinical criteria between drug responders and non-responders. So, the biological and technical biases were controlled by the following ways. 1) The systematic integration of gene expression profiles from multiple sources, meta-analysis, were used to analyze three datasets for reducing platform-caused batch effects. 2) All of samples from three datasets belongs to blood-related mononuclear cells, which are key cells lesion in CML disease. 3) Both of selected drugs, Imatinib and Dasatinib, directly bind BCR-ABL kinase and inhibit the function of BCR-ABL in CML therapy, which reduced drugs-caused bias. 4) All datasets used CR as major clinical criteria to decide drug response vs. non-response groups. The slightly difference in each dataset is that GSE2535 defined drug-response patient when they achieved CR within 9 months whereas GSE14671 defined them when they achieved CR within 12 months. This approach could reduce the heterogeneity of various datasets having a similar purpose and make them comparable to each other. To our knowledge, there is no other study that used the meta-analysis and classification analysis on gene expression dataset to identify a set of gene signatures and their biological functions for the response of TKI-based therapy in CML patients. Our study suggests potential drug-response biomarkers from gene expression profiles and provides a leading view to understand more precise control mechanisms of drugs.

## Additional files

Additional file 1: Table S1. Lists of differentially expressed genes of each dataset (GSE1467, GSE2535, and GSE33224). (XLS 115 kb) Additional file 2: Table S2. Lists of functional annotation terms of differentially expressed genes of each dataset (GSE14671, GSE2535, and GSE33224). (XLS 143 kb)
